# Individual and cumulative health afflictions are associated with greater impairment in physical and mental function in former professional American style football players

**DOI:** 10.1002/pmrj.12581

**Published:** 2021-05-13

**Authors:** Adam S. Tenforde, Bryan Cortez, Elaine Coughlan‐Gifford, Rachel Grashow, Jillian Baker, Aaron L. Baggish, Alvaro Pascual‐Leone, Lee M. Nadler, Frank E. Speizer, Herman A. Taylor, Marc G. Weisskopf, Ross Zafonte

**Affiliations:** ^1^ Department of Physical Medicine and Rehabilitation Spaulding Rehabilitation Hospital Charlestown Massachusetts USA; ^2^ Football Players Health Study at Harvard University, Harvard Medical School Boston Massachusetts USA; ^3^ Department of Environmental Health Harvard T.H. Chan School of Public Health Boston Massachusetts USA; ^4^ Cardiovascular Performance Program, Massachusetts General Hospital, Corrigan Minehan Heart Center Boston Massachusetts USA; ^5^ Hinda and Arthur Marcus Institute for Aging Research and Center for Memory Health, Hebrew SeniorLife Boston Massachusetts USA; ^6^ Department of Neurology Harvard Medical School Boston Massachusetts USA; ^7^ Guttmann Brain Health Institut, Institut Guttmann Universitat Autonoma Camí de Can Ruti Barcelona Spain; ^8^ Department of Medical Oncology Dana‐Farber Cancer Institute Boston Massachusetts USA; ^9^ Channing Division of Network Medicine Brigham and Women's Hospital Boston Massachusetts USA; ^10^ Cardiovascular Research Institute Morehouse School of Medicine Atlanta Georgia USA

## Abstract

**Background:**

Former American style football players (ASF players) have recognized health concerns associated with prior sport participation. It remains unknown whether categorizations of current health conditions, referred to in this report as afflictions (conceptually framed as neurocognitive, cardiovascular, cardiometabolic, sleep apnea, and chronic pain) are associated with physical and mental function.

**Objective:**

To evaluate the association of afflictions to physical and mental function. It was hypothesized that former National Football League players with any affliction would have worse function compared to unafflicted participants. It was anticipated that multiple afflictions would result in cumulative loss of function.

**Design:**

Cross‐sectional retrospective design.

**Setting:**

Academic medical multisite hospital system.

**Participants:**

A total of 3913 of 15,611 former ASF players who played professionally from 1960 to 2019 (response rate 25%).

**Assessment of Risk Factors**

Self‐report survey.

**Main Outcome Measures:**

Each participant completed the Patient Reported Outcomes Measurement Information System (PROMIS) Global Health Scale and Physical Function questionnaires. Responses were used to generate two physical function and one mental function subscale scores. Raw scores were converted to T‐scores categorized as impaired (T‐score < 40) or unimpaired (T‐score ≥ 40). Primary analyses measured the association of affliction to function (impaired or unimpaired).

**Results:**

After adjusting for confounders (age, race, position, number of seasons, age of first exposure to football, alcohol use, smoking history, and current body mass index), each affliction was associated with reduced physical function on the Global physical function subscale (risk ratio [RR] = 1.23‐2.45, all *P* < .005), physical function scale (RR = 1.24‐2.75, all *P* < .01), and mental function scale (RR = 1.34‐2.87, all *P* < .001), except that cardiovascular affliction was not associated with mental function (RR = 1.15, *P* = .15). The lowest functional measures were observed in those afflicted by chronic pain. Cumulative afflictions were associated with worse function.

**Conclusions:**

Afflictions are associated with cumulative reduction of function. Research evaluating how afflictions interact may help elucidate mechanisms for illness and develop interventions to optimize function.

## INTRODUCTION

Despite the popularity of American style football (ASF), the players, public, and scientific community have increasingly become concerned regarding the health status of former and current ASF players.^1^, ^2^ Study of the long‐term health effects and quality of life of former ASF players has primarily focused on neuropsychiatric disease.^3^, ^4^, ^5^, ^6^ Although it is important to understand the impact of a professional career on long‐term cognitive function, a large portion of former players are also diagnosed with or at risk for sleep disordered breathing,^7^, ^8^ obesity and high body mass index (BMI),^9^, ^10^ total knee and hip arthroplasties,^11^ cardiovascular disease,^12^, ^13^ and chronic pain^14^, ^15^ at rates higher than the general population. Previous studies^10^, ^16^ have proposed categorizing various related medical conditions into five “afflictions” and provided specific definitions for each affliction (e.g., cardiovascular disease is defined within a former ASF player as having one or more condition of prior myocardial infarction, prior stroke, or prior coronary revascularization intervention). Using these definitions, afflictions have been studied in former ASF players and used to understand association of characteristics to these health outcomes. For example, weight gain during participation in ASF is associated with greater rate of later life afflictions.^10^ How such afflictions are related to possible brain impact or other injuries sustained over a career in ASF remains poorly understood. However, they are likely to have an impact on overall health, well‐being, and activities of daily living.

Self‐reported cognitive impairment in former ASF players has been associated with depression, anxiety, attention deficit hyperactivity disorder, and worse functional status quantified using Patient Reported Outcomes Measurement Information System (PROMIS) physical and cognitive health scales.^13^ The limited current research on physical or mental function in former National Football League (NFL) players is primarily disease specific including concussion, neurodegenerative disease,^17^, ^18^ and early‐onset osteoarthritis^19^, ^20^ and limited to small sample sizes. Although comorbidities such as obesity, cardiovascular disease, and sleep apnea may influence overall health, the relative and cumulative effects of afflictions have not been fully explored. Understanding relative and cumulative effects of affliction on function could inform clinical approaches to optimize care in ASF players.^10^

The purpose of this study is to understand the effects of individual and combined afflictions on function of former professional ASF players using PROMIS physical and cognitive health scales. To achieve this aim, we analyzed data from a sample of former NFL players to compare their reported cognitive and physical function across previously described five common clinical health afflictions: cardiovascular disease, cardiometabolic disease, neurocognitive impairment, sleep apnea, and chronic pain. We hypothesized that afflictions in former NFL players will each be independently associated with a greater degree of impaired physical and mental function. Former NFL players with a greater number of afflictions were expected to have worse functional outcome.

## METHODS

### Study design

This study used a cross‐sectional, retrospective design to describe a cohort of former ASF players using self‐report completion of a survey. The Football Player Health Study at Harvard University (FPHS) is a multidisciplinary study focused on quantifying the health of former NFL players.^21^ Former NFL players who played professionally since 1960 were eligible to participate (active players were excluded) and were recruited using contact information provided by the National Football League Players Association and verified through cross‐referencing Pro Football Reference 22 to ensure their qualifications to participate. A 76‐item questionnaire was sent to all former players with contact information using both email and mailing address. Participation was optional, and no compensation was offered. Responses were collected electronically using REDCap (Vanderbilt, Nashville, TN)^23^, ^24^ or paper surveys using Scantron (Scantron Corporation, Tustin, CA). This study was approved by the Institutional Review Board at Beth Israel Deaconess Medical Center (Boston, MA).

### Self‐reported football exposure and other measures

On the survey former players were asked to indicate the positions that they most often played professionally (Table 1). Response options included offensive line, defensive line, linebacker, defensive back, running back, wide receiver, tight end, quarterback, kicker/punter, and special teams. The reference category was kicker/punter. As nearly every player who played special teams also listed an offensive or defensive position, we created an additional variable, similar to those done by other studies,^25^ to indicate participation in special teams, in three levels: none; “special strength” for players who also played offensive or defensive line or tight end; and “special speed” for players who also played running back, wide receiver, defensive back, or linebacker.

**TABLE 1:** Cohort characteristics

Variable	N	Percentage
Age (y)		
<40	951	24.3
40‐59	1626	41.6
60+	1336	34.1
Race		
Black	1463	37.4
White	2290	58.5
Other	112	2.9
Missing	48	1.2
Position		
Kicker	127	3.3
Offensive line	729	18.6
Defensive line	425	10.9
Linebacker	410	10.5
Special_speed	155	4.0
Special_strength	201	5.1
Defensive back	569	14.5
Running back	370	9.5
Tight end	303	7.7
Wide receiver	404	10.3
Quarterback	186	4.8
Special teams only	34	0.9
Age of first football exposure		
Less than age 12	1631	41.7
Age 12 or older	2232	57.0
Missing	50	1.3
Number of seasons		
1–6	2055	52.5
7+	1857	47.5
Missing	1	
Alcohol (drinks/week)		
None	1240	31.7
1–7	1454	37.2
8–14	694	17.7
15+	480	12.3
Missing	45	1.2
Smoking		
Never	3232	82.6
Past	521	13.3
Current	125	3.2
Missing	35	0.9
Body mass index category		
<25.0	211	5.4
25.0‐30.0	1627	41.6
30.0+	2047	52.3
Missing	28	0.7

*Note:* Presents participant demographics by age, race/ethnicity, primary playing position, alcohol, tobacco use, body mass index category at time of survey, and exposure (including age of onset and duration of participation).

Participants were asked, “How many seasons did you actively practice or play professional football?” Additionally, the first and last calendar years of professional play were queried. One participant was missing seasons of play and was excluded in analyses that used this variable.

Participants were asked, “How old were you when you began to play organized football?” We dichotomized age at first play as <12 or ≥12 years based on prior research.^17^ Fifty participants were missing age at first play. These participants were included in analyses as “missing.”

Alcohol use was measured by the number of alcoholic beverages (i.e., 12 oz of beer, 5 oz of wine, or one shot [2 cL] of alcohol, such as gin, rum vodka, etc.) consumed weekly. The measure was created based on two questions that asked about (1) the number of days a participant drank alcoholic beverages during a typical week and (2) the number of drinks consumed on a typical day that they drank. Forty‐five participants were missing response to alcohol use and were included as a separate “missing” category.

Tobacco use was queried by asking, “Have you smoked 20 packs of cigarettes or more in your lifetime?” Response options included “No”, “Yes, I currently smoke”, and “Yes, I smoked in the past.” Thirty‐five participants were missing tobacco use and were included as “missing.”

BMI was calculated from participant's self‐reported weight at time of survey and height and categorized as BMI < 25.0, 25.0 ≤ BMI < 30.0, or BMI ≥ 30.0. Twenty‐eight participants were missing either weight or height and were thus categorized as “missing.”

Participants were asked to self‐report race/ethnicity from the following choices: Black/African American, Native Hawaiian/Pacific Islander, White, Asian, American Indian/Alaskan Native, Other. From available responses, race was coded as Black, White, or Other. Forty‐eight participants were missing race and included as “missing.”

### Definition of affliction

Clinical afflictions were defined based on five domains using health information from patient self‐report, using similar criteria as previously reported^10^: neurocognitive impairment, cardiovascular disease, sleep apnea, cardiometabolic disease, and chronic pain. For neurocognitive impairment, the survey asked if a health care provider had ever given the participant a diagnosis of dementia or chronic traumatic encephalopathy or if a former player was currently taking medication to treat memory loss. Cardiovascular disease was queried on the survey by asking former players if they have ever received a clinician‐generated diagnosis of myocardial infarction, stroke, or cardiac revascularization interventions (bypass surgery, angioplasty, or stent). Sleep apnea was queried by asking former players if a health care provider had ever given a diagnosis of sleep apnea. Cardiometabolic disease was defined by asking respondents if they were diagnosed and currently taking medications for hypertension, hyperlipidemia, and diabetes mellitus. Finally, chronic pain responses were collected by asking if they were currently prescribed medications for pain. These five binary outcome variables were designed to define afflictions across the most common domains of interest for the study sample (Table 2).

**TABLE 2:** Distribution and definition of affliction and outcome variables

Variable	N	Percentage	Description
Afflictions			
Neurocognitive	275	7.0	Self‐reported clinician‐generated diagnosis of dementia or chronic traumatic encephalopathy OR current prescription of medication to treat memory loss
Cardiovascular	346	8.8	Self‐reported clinician‐generated diagnosis of prior myocardial infarction OR stroke OR coronary revascularization intervention (bypass surgery, angioplasty, or stent)
Sleep apnea	869	22.2	Self‐reported clinician‐generated diagnosis of sleep apnea
Cardiometabolic	616	15.7	Current prescription of at least two medications for hypertension, diabetes, or high cholesterol
Chronic pain	1061	27.1	Current prescription for pain medication
Number of afflictions			
0	1940	49.6	
1	1134	29.0	
2	551	14.1	
3+	288	7.4	
PROMIS global mental function			
Unimpaired	2569	67.4	T‐score is no worse than one SD below the mean (T = 40+)
Impaired	1242	32.6	One or more SDs below the mean (T < 40)
PROMIS global physical function			
Unimpaired	2789	73.3	T‐score is no worse than one SD below the mean (T = 40+)
Impaired	1016	26.7	One or more SDs below the mean (T < 40)
PROMIS physical function 6b scale			
Unimpaired	2808	71.9	T‐score is no worse than one SD below the mean (T = 40+)
Impaired	1099	28.1	One or more SDs below the mean (T < 40)

*Note:* Displays the number and percent of each affliction and measures of two physical function and one mental function subscale. T‐scores present standard values to reference population with a value T‐score of 50 reflecting average function with standard deviation (SD) of 10. A T‐score <40 was defined as impaired function.

Abbreviation: PROMIS, Patient‐Reported Outcomes Measurement Information System.

### Physical and mental health outcomes

Within the initial survey the former players completed a series of standardized questionnaires: PROMIS Physical Function v2.0 ‐ Short Form (6b) and PROMIS Global Health Scale v1.2. PROMIS Physical Function queries perceived difficulty of completing daily physical tasks and chores including vacuuming, yard work, and house cleaning. Response options for perceived difficulty to completing a task range from 0: “without any difficulty” to 4: “unable to do.” PROMIS Global Health Scale v1.1 includes a four‐question subset, global mental health, that addresses mental health and function. These items query perceived mental health including mental function, emotional problems, quality of life, and social discretion. Response options range from 0: “poor” to 4: “excellent” and focus on how respondents rate their overall mental health. PROMIS Global Health Scale v1.1 includes a four‐question subset, global physical health, that address physical health, physical function, pain, and fatigue. Response options range from 0: “poor” to 4: “excellent” and focus on how respondents rate their overall physical health. These questionnaires have been validated and tested across various diseases and disorders including depression,^26^ chronic pain,^27^ anterior cruciate ligament reconstruction,^28^ and chronic heart failure.^29^

### Statistical analysis

The PROMIS Physical Function, Global Health ‐ Physical Health subset, and Global Health ‐ Mental Health subset raw scores for each participant were converted to a T‐score. The T‐score represents normalized values of total raw score for each subscale standardized to a large sample of the general population. A T‐score of 50 represents mean performance and SD is 10. A lower T‐score represents worse function. The strength of association of each T‐score with each medical condition within an affliction (e.g., for cardiometabolic affliction, association of each component hypertension, hyperlipidemia, and diabetes mellitus) was evaluated using a ranked point biserial correlation. We evaluated the T‐score values conservatively using a categorical variable of impaired (T‐score < 40) and unimpaired (T‐score ≥ 40) for those with a given affliction and by number of afflictions. We estimated multivariate‐adjusted risk ratios and confidence intervals using a Poisson regression with robust error variance.

The model controlled for factors previously shown to influence quality of life function including age, primary playing position, duration of play, initial age of participation, tobacco and alcohol use,^6^, ^17^ and BMI. Statistical analyses were conducted in SAS (SAS v. 9.4, SAS Institute. Cary, NC).

## RESULTS

### Cohort characteristics

The participants in this study included 3913 former players who completed the survey (total surveyed at time of data analysis 15,611, response rate 25.1%). Former players were primarily Caucasian and one‐third were above age 60. A majority of participants reported age of onset in participation in ASF of 12 years of age or older, and fewer than half of the cohort completed seven or more seasons in the NFL (Table 1).

### Prevalence of afflictions

**FIGURE 1:**
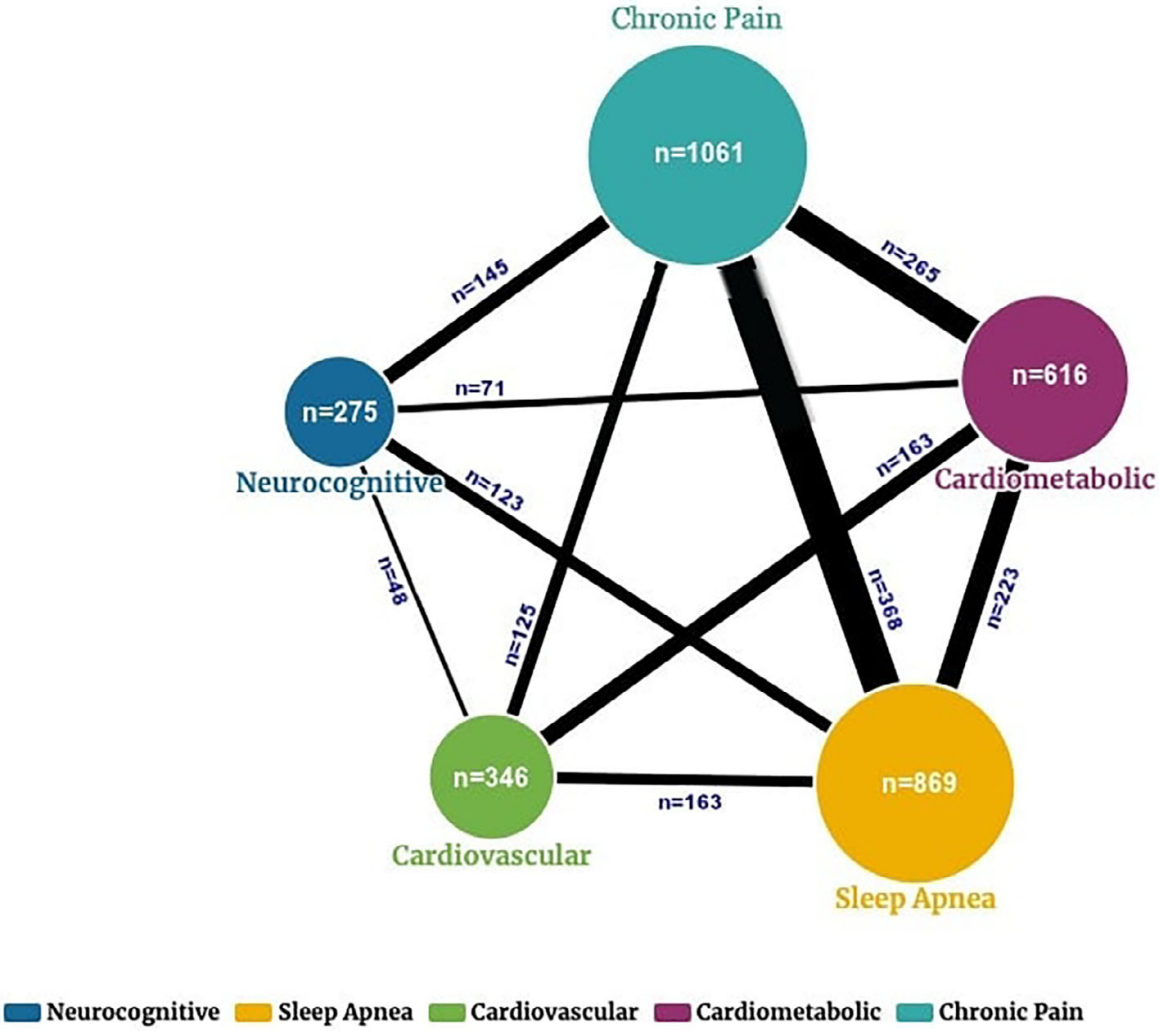
Displays the number of individuals by affliction and the overlap of players with afflictions. Larger size circles reflects relative number with an affliction, and bolder lines suggest larger proportion of overlap for an affliction. Notably former players were most likely to meet affliction criteria for chronic pain and lowest number met criteria for neurocognitive affliction

A majority of participants reported one or more afflictions (Figure 1). Overall, 29% of all participants (n = 1134) reported one affliction, whereas 14% (n = 551) had two afflictions, and 7.4% (n = 288) had three of more afflictions (Table 2). Chronic pain was the most common affliction (27.1%), followed by sleep apnea (22.2%), cardiometabolic disease (15.7%), cardiovascular disease (8.8%), and neurocognitive impairment (7.0%).

### Prevalence of functional impairment within cohort

The mean T‐score for each functional scale was below the normalized T‐score of 50 for all scales, including global mental function (45.53 ± 9.18), global physical function (46.43 ± 10.21), and the six‐question physical function (46.35 ± 8.85). One‐third of participants (n = 1016, 26.7%) met criteria for impairment in mental function defined as T‐score < 40. Over one‐quarter of all participants met criteria for impairment in global physical function (n = 1242, 32.6%) or physical function 6b measure (n = 1099, 28.1%).

### Association of affliction to function

Both cognitive and physical functional subscale scores were associated with medical conditions that comprised the afflictions, with the exception of nonsignificant associations for mental function to prior myocardial infarction, cardiac surgery, and current hyperlipidemia (Table 3). As expected, older mean age was observed to be associated with a greater number of afflictions; those with no afflictions were youngest (mean age ± SD: 47.5 ± 13.5 years old) whereas the highest prevalence of three of more afflictions was observed in the oldest participants (62.0 ± 11.8 years old, *P* < .001). There were no differences in number of afflictions by race (data not shown). There were no differences in number of afflictions by age of first exposure to football, total number of seasons, or alcohol intake.

**TABLE 3:** Correlations between individual affliction elements and functional outcome scores using rank point biserial correlation analysis

	Rank point Biserial correlation										
	Dementia	Chronic traumatic encephalopathy	Med. for memory loss	Heart attack	Stroke	Cardiac surgery	Hypertension	Diabetes	High cholesterol	Obstructive sleep apnea	Pain medication
Global mental T‐score	−0.21 <.001 3805	−0.19 <.001 3805	−0.22 <.001 3683	−0.03 .06 3805	−0.04 .02 3805	−0.02 .37 3666	−0.10 <.001 3750	−0.08 <.001 3706	0.00 .93 3702	−0.20 <.001 3805	−0.31 <.001 3680
Global physical T‐score	−0.19 <.001 3811	−0.16 <.001 3811	−0.19 <.001 3686	−0.10 <.001 3811	−0.08 <.001 3811	−0.08 <.001 3673	−0.22 <.001 3753	−0.15 <.001 3713	−0.10 <.001 3707	−0.25 <.001 3811	−0.43 <.001 3680
Physical function T‐score	−0.21 <.001 3907	−0.17 <.001 3907	−0.19 <.001 3773	−0.11 <.001 3907	−0.10 <.001 3907	−0.10 <.001 3758	−0.24 <.001 3841	−0.15 <.001 3798	−0.14 <.001 3792	−0.24 <.001 3907	−0.42 <.001 3766

*Note:* Displays rank point biserial correlation of individual disease states that are used to define affliction. Dementia, chronic traumatic encephalopathy, and current prescription for memory loss are each used to define neurocognitive affliction. Prior heart attack, stroke, or cardiac surgery define cardiovascular affliction. Cardiometabolic affliction is defined as two of three conditions of diabetes, hypertension and high cholesterol. Sleep apnea is defined as prior diagnosis of sleep apnea. Pain affliction is defined as current prescription to treat pain. The strength of association to each functional outcome, *P* value of statistical significance, and number of total response for observations are displayed and separated by each functional outcome.

Accounting for potential confounders (age, race, position in ASF, age of first exposure to football, number of seasons, BMI, alcohol and tobacco use), each affliction was associated with higher likelihood of both impaired physical and mental function, with exception of cardiovascular affliction (Table 4). Further, a greater number of afflictions was associated with greater likelihood of impaired function. Compared to those without affliction, participants with one affliction were at twofold increased risk for impaired mental and physical function, and participants with two and three afflictions at nearly three‐ and fivefold increased risk, respectively.

**TABLE 4:** Fully adjusted model includes age, race, position, number of seasons, age of first exposure, alcohol, smoking history, and BMI category at time of survey

Affliction	Mental function (4 Q)		Physical function (4 Q)		Physical function (6 Q)	
	N = 3804		N = 3810		N = 3906	
	N_missing_ = 109^a^		N_missing_ = 103^a^		N_missing_ = 7	
	RR (95% CI)	*P* value	RR (95% CI)	*P* value	RR (95% CI)	*P* value
Neurocognitive	2.87 (2.58, 3.19)	<.001	1.98 (1.78, 2.19)	<.001	2.28 (2.02, 2.58)	<.001
Cardiovascular	1.15 (0.95, 1.39)	.154	1.23 (1.07, 1.40)	.003	1.24 (1.05, 1.45)	.009
Cardiometabolic	1.34 (1.16, 1.54)	<.001	1.43 (1.29, 1.59)	<.001	1.35 (1.19, 1.54)	<.001
Sleep apnea	1.65 (1.48, 1.84)	<.001	1.63 (1.49, 1.79)	<.001	1.76 (1.57, 1.96)	<.001
Chronic pain	2.13 (1.92, 2.35)	<.001	2.45 (2.25, 2.68)	<.001	2.75 (2.46, 3.06)	<.001
Number of afflictions						
0	Reference		Reference		Reference	
1	2.01 (1.76, 2.31)	<.001	2.25 (1.98, 2.56)	<.001	2.52 (2.14, 2.98)	<.001
2	2.60 (2.24, 3.02)	<.001	3.18 (2.78, 3.64)	<.001	3.69 (3.10, 4.38)	<.001
3+	4.15 (3.55, 4.86)	<.001	4.14 (3.60, 4.76)	<.001	5.08 (4.24, 6.10)	<.001

*Note:* Displays risk ratios for a given affliction meeting criteria of impaired function (T‐score < 40). Each affliction, with the exception of cardiovascular affliction, was associated with higher risk of mental function impairment. All afflictions were associated with higher risk for physical function impairment for both physical function subscales. Compared to former players with no afflictions, greater number of afflictions was associated with cumulative elevated risk for meeting impaired function (T‐score < 40).

^a^ Total number of seasons could not be obtained for one participant.

Abbreviations: BMI, body mass index; CI, confidence interval; RR, relative risk.

## DISCUSSION

The purpose of this study was to evaluate the association of previously described affliction to physical and mental function in a population of former ASF players. As expected, former NFL players with a given affliction reported worse functional status. The effects of multiple afflictions may be cumulative as former NFL players with greater number of afflictions reported greater impairments in physical and mental function. The concept of afflictions used in the current and prior reports^10^, ^16^ may provide a framework to understand health in this athlete population. Findings from this report of former ASF players having multiple afflictions and how afflictions can affect function may help guide exploration on bridging topics; for example, the role of cumulative sports‐induced inflammation of the musculoskeletal and neurological systems could expand our understanding for mechanisms and treatment of neuroinflammation on widespread musculoskeletal pain.^30^ Applying the concept of affliction to guide treatment requires consideration beyond neurocognitive effects of sport and invites further discussion of treating possible contributing factors for impaired function that optimize care models.

Physical function was negatively associated with all afflictions and mental function was lower for most afflictions (excluding cardiovascular) and for most medical conditions within a given affliction domain. These findings expand on prior observations in former ASF players and validate observations that afflictions in this population often impair function. Worse mental function for former ASF players with neurocognitive disease (including self‐reported clinician‐generated diagnosis of dementia, memory loss, and self‐reported diagnoses for chronic traumatic encephalopathy) and prior stroke is consistent with prior reports.^5^, ^6^ However, pain affliction had the highest negative correlation to mental function. Furthermore, individuals with hypertension and diabetes also had impaired mental function. Although the study design limits understanding the mechanisms for these associations, the results suggest that afflictions outside neurodegenerative disease should be considered to also affect mental function.

Pain affliction, as defined by current prescription of pain medication, had the strongest association with both impaired mental and physical function. Our report highlights the importance of more effectively treating pain in this population. The type of medication prescribed cannot be determined based on study design. However, prior reports suggest a majority of former ASF players may have a prior prescription for opioids during active playing years with a concerning “misuse” rate as high as 71% during active playing years.^18^ This behavior was observed as associated with anxiety and depression, and prior prescription of opioids during active playing years was associated with 30% higher risk for current use.^31^ Opioid use for chronic pain has been associated with worse physical and mental function in other populations^32^ but the cross‐sectional design of these investigations is prone to bias and reverse causality. Further, centralized pain through mechanisms of neuroinflammation resulting from proinflammatory central cytokines and chemokines has been implicated in widespread pain.^30^ The interactions of pain, mood disorders, and insomnia have been postulated to link to neurobiological mechanisms including disruption to the mesolimbic dopaminergic system.^33^ The mechanism for how pain influences both physical and mental function in this population is poorly characterized. Given the association of lowest functional measures in those with the chronic pain affliction, we suggest understanding mechanisms for pain to guide novel treatment strategies in this population should be a priority.

There are important clinical implications of our findings. Clinicians charged with the care of former ASF athletes will be optimally effective when they consider the interdependent and additive associations between conventional disease processes and functional impairment. Specifically, clinicians encountering former ASF athletes with one or more of the key afflictions examined in this study are encouraged to screen for concomitant functional impairment. Similarly, former ASF athletes presenting with functional impairment should be screened for disorders of sleep, chronic pain, neurocognitive impairment and cardiometabolic/cardiovascular disease. In both situations, optimal care likely integrates the combination of guideline‐based therapeutic interventions to treat affliction coupled with concomitant interventions geared toward improving functional status. The latter may best be addressed with the combination of targeted lifestyle intervention and a team‐based care paradigm with expertise in physical therapy, occupational therapy, and mental health. Studies designed to establish causal relationships between affliction and functional impairment with an emphasis on defining optimal treatment strategies to address both sides of this equation represent logical future work.

## LIMITATIONS

There are limitations to the results presented in this study. Although this is the largest sample of former ASF players studied to date, we cannot confirm this sample is representative of all former NFL players given the response rate. The definitions for each disease and affliction were limited to prior self‐reported clinician‐generated diagnosis of a condition or prescription of medications. To define an affliction, we used current prescription of medication in contrast to Churchill et al.^10^ Our definition contributed to a lower proportion of former ASF players meeting criteria for affliction in neurocognitive and cardiometabolic categories than reported by Churchill. However, this approach was chosen as a more conservative approach to measuring the association of a current affliction to function at time of survey completion. The definitions of afflictions used for this study may be conservative and potentially under report the true prevalence of some afflictions (e.g., former ASF players with chronic pain may not seek professional treatment). Although using a validated metric of physical and mental function, the study does not provide a control group; identifying an appropriate population for comparison would help substantiate our findings. The cross‐sectional design impedes our ability to identify the mechanism both within and between affliction domains to function; longitudinal investigations including ongoing prospective data collection may help uncover how afflictions alone and in concert interact to cause functional impairments and define disease mechanisms to optimize treatment. Future studies on this population of ASF players using objective measures may elucidate the complex and interdependent pathways to disease.

## CONCLUSIONS

In summary, the most afflicted former NFL players reported worse physical and mental function. The pain affliction had the strongest association with impaired physical and mental function. The findings suggest a deeper understanding of the complex and interconnected concept of afflictions is required to understand the health of former NFL players and when applied correctly has opportunity to improve function and quality of life in this population.

## AUTHOR CONTRIBUTIONS

Adam S. Tenforde, Rachel Grashow, Aaron Baggish, Alvaro Pascual‐Leone, Lee M. Nadler, Frank E. Speizer, Herman A. Taylor, Marc G. Weisskopf, and Ross Zafonte were responsible for conception and design of the work. Adam S. Tenforde, Bryan Cortez, Elaine Coughlan‐Gifford, Rachel Grashow, Jillian Baker and Ross Zafonte were responsible for analysis and interpretation of the data. All authors were responsible for drafting and revising the manuscript and provided final approval of the version to be published. All authors agree to be accountable for all aspects of the work in ensuring that questions related to the accuracy or integrity of any part of the work.

## DISCLOSURES

Dr. Baggish reports funding from the National Institute of Health/National Heart, Lung, and Blood Institute, the National Football Players Association, and the American Heart Association and receives compensation for his role as team cardiologist from US Soccer, US Rowing, the New England Patriots, the Boston Bruins, the New England Revolution, and Harvard University. Dr. Zafonte receives royalties from (1) Oakstone for an educational CD, Physical Medicine and Rehabilitation a Comprehensive Review; (2) Demos publishing for serving as co‐editor of the text *Brain Injury Medicine*. Dr. Zafonte serves on the Scientific Advisory Board of Oxeia. Biopharma, Biodirection, ElMINDA, and Myomo. He also evaluates patients in the MGH Brain and Body‐TRUST Program which is funded by the NFL Players Association. Dr. Zafonte was partially supported by NIDILRR: 90DP0039‐03‐00, 90SI5007‐02‐04,90 D P0060; USAMRC‐W81XWH‐112‐0210, NIH: 4 U01NS086090‐04; 5R24HD082302‐02;5U01NS091951‐03 and serves a co‐principal investigator on a National Institute of Neurological Disorders and Stroke funded T‐32 on neurorehabilitation and Neurotechnology. He is principal investigator on a grant entitled the Football Players Health Study at Harvard University, which is funded by the NFL Players Association (NFLPA). Dr Zafonte evaluates patients for the MGH brain and Body TRUST center sponsored in part by the NFLPA and serves on the Mackey White health committee. Dr. Tenforde has no disclosures related to this work. He serves as senior editor for PM&R Journal. He gives professional talks such as grand rounds and medical conference plenary lectures and receives honoraria from conference organizers. He has participated in research funded by The Arnold P. Gold Foundation (physician and patient care disparities) and American Medical Society for Sports Medicine (bone density research). All authors report prior or current funding support from the Football Player Health Study at Harvard University.

**CME Question**

Which affliction domain was associated with a negative impact on physical function in American style football players but did not show a negative correlation with mental function?

 a. Sleep apnea

 b. Cardiovascular

 c. Neurocognitive

 d. Cardiometabolic

**Answer online at** https://onlinelearning.aapmr.org/
